# Variability of contact process in complex networks

**DOI:** 10.1063/1.3664403

**Published:** 2011-12-07

**Authors:** Kai Gong, Ming Tang, Hui Yang, Mingsheng Shang

**Affiliations:** 1 Web Sciences Center, University of Electronic Science and Technology of China, Chengdu 610054, People’s Republic of China; 2 2Computer Experimental Teaching Center, University of Electronic Science and Technology of China, Chengdu 610054, People’s Republic of China

## Abstract

We study numerically how the structures of distinct networks influence the epidemic dynamics in contact process. We first find that the variability difference between homogeneous and heterogeneous networks is very narrow, although the heterogeneous structures can induce the lighter prevalence. Contrary to non-community networks, strong community structures can cause the secondary outbreak of prevalence and two peaks of variability appeared. Especially in the local community, the extraordinarily large variability in early stage of the outbreak makes the prediction of epidemic spreading hard. Importantly, the bridgeness plays a significant role in the predictability, meaning the further distance of the initial seed to the bridgeness, the less accurate the predictability is. Also, we investigate the effect of different disease reaction mechanisms on variability, and find that the different reaction mechanisms will result in the distinct variabilities at the end of epidemic spreading.

The variability of outbreaks is defined as the relative variation of the prevalence. In order to assess the accuracy and the forecasting capabilities of numerical models, the variability of outbreaks has been investigated in many studies. In numerical models, many factors such as network structures, travel flows, and initial conditions can affect the reliability of the epidemic spreading forecast. Recently, a contact process model with identical infectivity is proposed to study both dynamical processes and phase transitions of epidemic spreading in complex networks, but the predictability of the model is totally overlooked. In this paper, by investigating the variabilities of contact process in distinct networks, we show numerically that the bridgeness plays a significant role on the predictability of the epidemic pattern in community network, meaning the further distance of the initial seed to the bridgeness, the less accurate the predictability is. Hopefully, this work will provide us further understanding and new perspective in the variability of contact process in complex networks.

## I. INTRODUCTION

The great threat of epidemic spreading to human society has been strongly catching scientists’ eyes.^1,2^ In order to realize the impact of diseases and develop effective strategies for their control and containment, the accurate mathematical models of epidemic spreading are the basic conceptual tools.^1–4^ In mathematical models, the dynamical patterns of epidemic spreading will be influenced by many different factors such as the age and social structure of the population, the contact network among individuals, and the meta-population characteristics.^5^ Especially, the heterogeneity of the population network^6^ can result in the absence of endemic threshold when the population size is infinite and the exponent of degree distribution *γ* ≤ 3.^7–11^ With the further study, the local structures of complex networks (such as degree correlation, clustering coefficient, and community structure) bring quantitative influences on epidemic spreading.^12–14^ Considering the complicated local structures in real networks, the forecasting capabilities (i.e., variability) of current numerical models have been investigated.^15^ In addition, both the stochastic nature of travel flows,^16,17^ and initial conditions can affect the reliability of the epidemic spreading forecast.^18–24^

In view of this point, Colizza *et al.* have studied the effect of the airline transportation network on the predictability of the epidemic pattern by means of the normalized entropy function^18^ and found that the heterogeneous distribution of this network contributes to enhancing the predictability. In complex networks, many factors can decrease the forecasting accuracy of epidemic spreading. Crépey *et al.* have found that initial conditions such as the degree heterogeneity of the seed show a large variability on the prediction of the epidemic prevalence, and the infection time of nodes have non-negligible fluctuations caused by the further distance and the multiplicity of paths to the seed.^19^ Comparing the scale-free network (SFN) with community structure^25–30^ with the random SFN, the predictability of the prevalence can be found to be better.^31^

The common assumption in all the aforementioned works is that each node’s potential infection-activity (infectivity), measured by its possibly maximal contribution to the propagation process within one time step, is strictly equal to its degree. However, there are still many real spreading processes which can not be described well by this assumption.^32^ Therefore, a contact process (CP) model with identical infectivity is proposed to study the epidemic spreading in complex networks.^33^ Almost all studies in CP are focused on dynamical processes and phase transitions,^32–42^ but the predictability of the model is totally overlooked. To this end, we study how the structures of distinct networks (i.e., homogeneous, heterogeneous, and community networks) influence the variabilities of epidemic patterns in CP. Through numerical experiments, we find that the community structures can remarkably influence the prevalence and its variability, contrary to non-community networks (i.e., homogeneous and heterogeneous networks). It is worth noting that it’s hard for the extraordinarily large variability in a local community to predict the epidemic prevalence.

This paper is organized as follows. In Sec. II, we briefly describe disease models in CP in complex networks and provide quantitative measurements of the predictability of epidemic spreading. In Sec. III, we investigate the prevalence variabilities in both random graph (RG)^43^ and SFN (Ref. 44). In Sec. IV, we discuss the essential differences of the prevalence variabilities both in the global network and the local community. Finally, we draw conclusions in Sec. V.

## II. CP MODEL IN COMPLEX NETWORKS

In our model, three distinct networks, i.e., the homogeneous, heterogeneous, and community networks are adopted to investigate the predictability of epidemic spreading therein. First, as the mother of all network models, the random graph of Erdős and Rényi^43^ is regularly used in the study of complex networks because networks with a complex topology and unknown organizing principles often appear randomly.^45^ Random graph is defined as a graph with *N* nodes and connection probability *p*, which has a Poisson distribution. Second, since scale-free property is observed in many real complex systems, dynamics study on scale-free networks has been holding everyone’s concern. In 1999, Barabási and Albert (BA) put forward the most classical SFN model which is rooted in two generic mechanisms: growth and preferential attachment.^44^ As there are community structures in social networks, the last studied structure substrate is community network.^46^ Here we will adopt a simplified community model proposed by Liu and Hu, which emphasizes on the community feature in social networks.^25^ For simplicity, two independent random graphs are first produced, and then two RGs are connected randomly by only one link.

In general, the standard disease models conclude susceptible-infected (SI), susceptible-infected-susceptible (SIS), and susceptible-infected-refractory (SIR) epidemiological model. Each node of the network represents an individual, and each link plays as one connection which transmits disease to other node. In SI (SIS or SIR) model, “S,” “I,” and “R” represent, respectively, the susceptible (healthy), the infected, and the refractory (recovered) state. At each time step of contact process, each infected node randomly contacts one of its neighbors, and then the contacted neighboring node will be infected with probability *λ* if it is in the healthy state or else its state will stay the same. At the same time, each infected nodes is cured and becomes susceptible (refractory) with rate *μ* in SIS (SIR) model. To eliminate the stochastic effect of the disease transmission, we can set *λ* = 1 and *μ* = 0.2.

In order to analyze the effect of the underlying network topology on the predictability of epidemic spreading, the variability of outbreaks is defined as the relative variation of the prevalence [density of infected individuals *i*(*t*)] given by Ref. 19

$$\Delta [i(t)]=\frac{\sqrt{〈i{\left(t\right)}^{2}〉-{〈i(t)〉}^{2}}}{〈i(t)〉}.$$ (1)

Δ[i(t)]=0 denotes all independent dynamics realizations are essentially the same, and the prevalence in the network is deterministic. Larger Δ[i(t)] means worse predictability that a particular realization is far from average over independent realizations.

## III. PREDICTABILITY IN HOMOGENEOUS AND HETEROGENEOUS NETWORKS

**FIG. 1.**
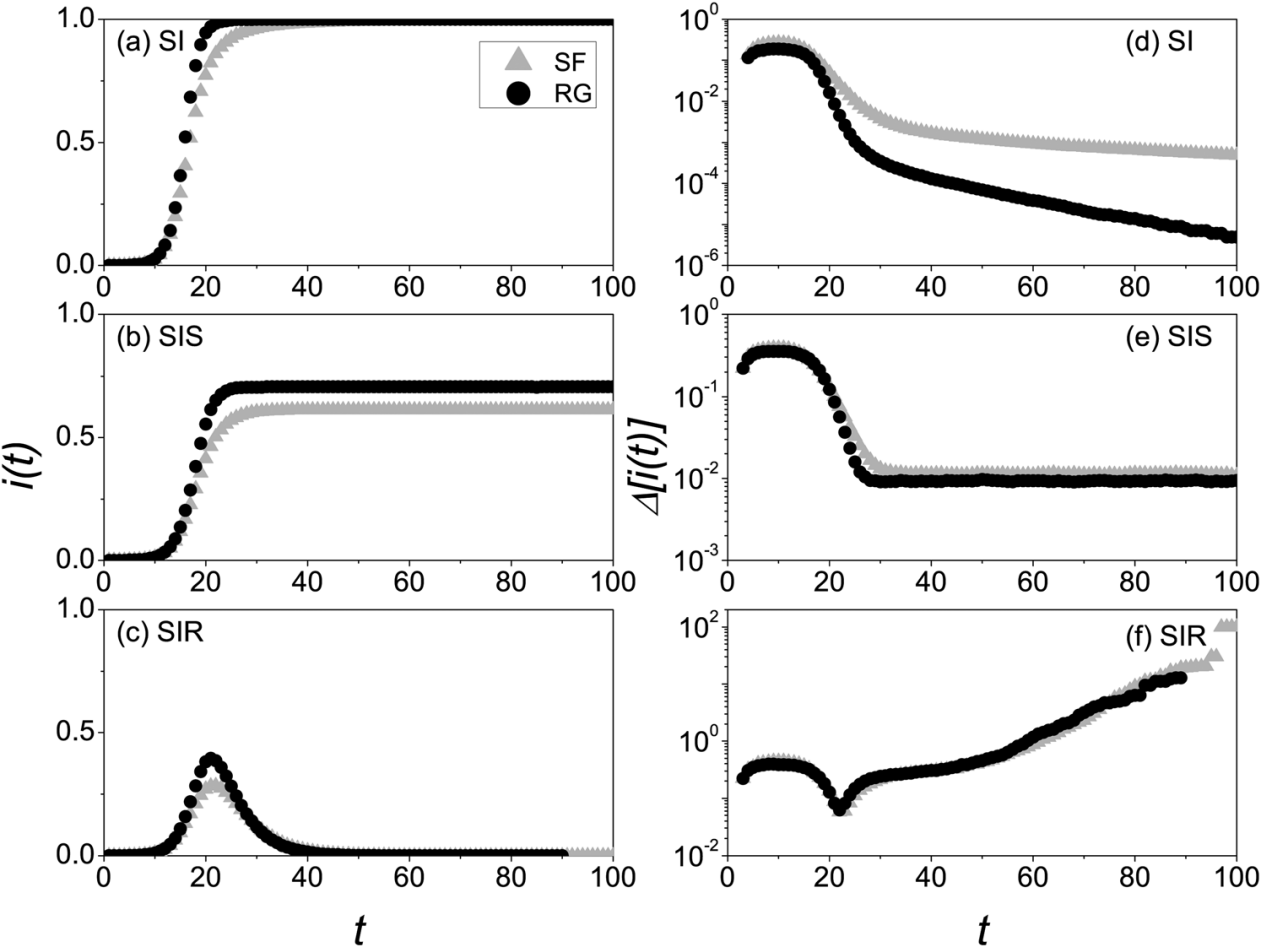
Evolution of both *i*(*t*) and Δ[i(t)] for the different disease models where the “triangles” and “circles” denote the cases of SF and RG networks with the random initial seeds. *i*(*t*) versus *t* for SI model (a), SIS model (b), and SIR model (c), and Δ[i(t)] versus *t* for SI model (d), SIS model (e), and SIR model (f). The parameters are chosen as *N* = 0.5 × 10^4^, 〈k〉=10, *λ* = 1, and *μ* = 0.2. The results are averaged over 2 × 10^4^ independent realizations in one network.

The first issue of our study is how the heterogeneity of network structures influences the variability of the prevalence in CP. By using a numerical approach in this section, we analyze the variabilities of outbreaks generated by different sets of initial nodes, both for random graphs and scale-free networks with the same network size and average degree. Considering the fact that the results of a particular network can be generalized to any instances of network model,^19^ the numerical simulations we studied here are run in one network. In Fig. 1, we show the curves *i*(*t*) and Δ[i(t)] computed for the different disease models in both RG and SFN. For SI model in Fig. 1(a), the density of infected *i*(*t*) in RG reaches its stationary state faster than that in SFN; for SIS model in Fig. 1(b), the stationary *i*(*t*) in RG is greater than that in SFN; for SIR model in Fig. 1(c), RG has the higher peak prevalence. Contrary to the results for the case of contacting all neighbors, it is first discovered that the heterogeneous structure can slow down the prevalence of outbreaks in CP. Because hubs may be contacted many times by their neighboring nodes at each time step, the total contact ability (i.e., the actual number of contacting nodes at one time step) of SFN is reduced further accordingly; as a result, the hub effect holds back the prevalence of diseases. Meaningfully, owing to the limited contact ability of CP, the infected densities starting from the initial infected nodes (seeds) with different degrees are almost the same in SFN, which is distinct from the results for the case of contacting all neighbors.^19^

As shown in Figs. 1(d)–1(f), there is slightly different between variabilities in RG and SFN when *t* < 20, which implies heterogeneous structure does not visibly alter the predictability of CP before the outbreak of disease. An important contribution of this study is to analyze the differences among the variabilities of three kinds of disease models. From the comparison among them, we find that different recovery mechanisms can result in distinct variabilities at the end of epidemic spreading. For SI model in Fig. 1(d), the time arriving at *i*(*t*) = 1 varies in a mass of realizations, which can induce an exponential decay of the variability when *t* > 30. For SIS model in Fig. 1(e), the variability of the prevalence will keep on a steady value in stationary state. For SIR model in Fig. 1(f), due to the different lifetimes of the epidemics in a mass of independent realizations,^18^ the greater and greater variabilities are observed by approaching the end of the epidemics.

## IV. PREDICTABILITY IN THE GLOBAL NETWORK AND THE LOCAL COMMUNITY

### A. Global network

**FIG. 2.**
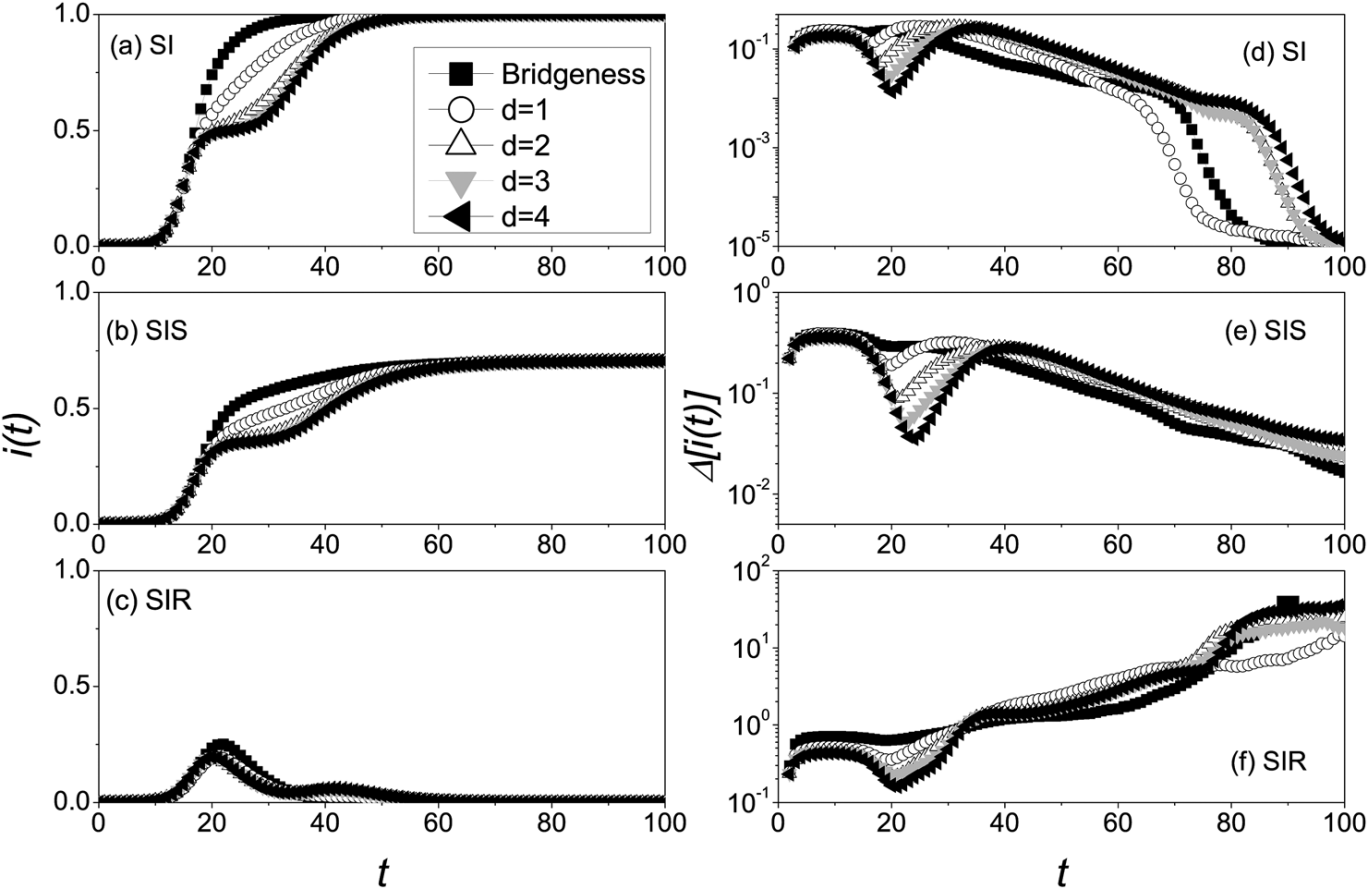
Evolution of both *i*(*t*) and Δ[i(t)] in community networks where the “squares,” “circles,” “triangleups,” “triangledowns,” and “trianglelefts” denote the cases of the bridgeness, *d* = 1, 2, 3, and 4, respectively. *i*(*t*) versus *t* for SI model (a), SIS model (b), and SIR model (c), and Δ[i(t)] versus *t* for SI model (d), SIS model (e), and SIR model (f). The parameters are chosen as *N* = 10^4^, 〈k〉=10, *λ* = 1, and *μ* = 0.2. The results are averaged over 2 × 10^4^ independent realizations.

As many social networks combined by several communities, such as Facebook,^47^ YouTube,^48^ and Xiaonei,^49^ information propagation taking place in community networks^25–29^ is one of the most important subjects studying in complex networks, but in CP, the related research has been ignored for a long time. Therefore, in this section, we study the variability of CP in a very simple community network, where two RGs are connected randomly by only one link. Obviously, this network has a strong strength of community structure. In order to normalize the terms of community network, we define the link as weak tie^50^ and call two nodes connected by this link “bridgeness.”^51^ We first investigate the time evolution of epidemics generated by different seeds staying away from the bridgeness, and it is noted that there is only one initial seed in each realization. From Figs. 2(a)–2(c), we can get that the closer the seed to the bridgeness, the epidemic spreads much faster in the global network, and among all cases, the epidemic starting at the bridgeness spreads fastest. For SI model, the further distance to the bridgeness such as *d* = 3, 4 induces two periods of the quickly rising trend at the beginning time *t* = 10, 30, respectively. If the initial seed is far away from the bridgeness, the disease will be restricted in the first community for a long time till the bridgeness infected, in which almost all nodes is infected. As a result, the outbreak in the second community just starts at that moment the prevalence in the first community get towards the end, which causes the second outbreak. In the case of SIS and SIR model, the recovery mechanism reduces this phenomenon occurred; for instance, there is the tiny second peak of the prevalence for *d* = 3, 4 in Fig. 2(c). From the above, it is found that the bridgeness plays a distinctly important role in the rapid transmission of information in CP.

**FIG. 3.**
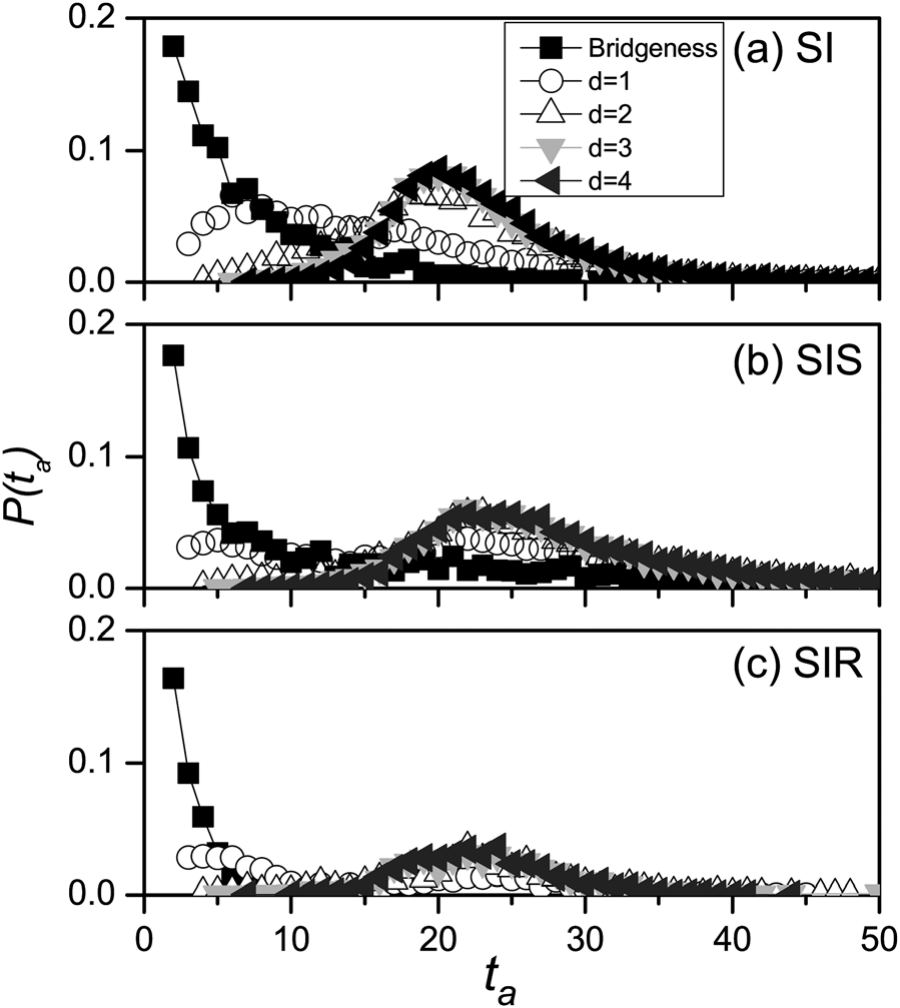
The distribution of arrival time of disease in the second community for SI model (a), SIS model (b), and SIR model (c), where the “squares,” “circles,” “triangleups,” “triangledowns,” and “trianglelefts” denote the cases of the bridgeness, *d* = 1, 2, 3, and 4, respectively. The results are averaged over 2 × 10^4^ independent realizations.

The variability of prevalence in community network is distinct from that in the network which has no community structure. As shown in Figs. 2(d)–2(f), the curves Δ[i(t)] display two peaks because of the time delay between two outbreaks occurred in different communities. In addition, the further distance to the bridgeness makes the second peak occur much later. In Fig. 2(d), for SI model, the first peak corresponds to the prevalence in the community with the initial seed, so the variability is almost the same as that in RG before *t* = 10. With the outbreak in the second community, the second peak occurs. Owing to the greater randomness of the time that disease first occurs in the second community (see Fig. 3), the second peak of the variabilities is slightly greater than the first peak. After the infection density is close to saturated at *t* ≈ 40 (see Fig. 2(a)), the variability will be on exponential decay. As all nodes of community network are infected in more and more realizations, the variability Δ[i(t)] will rapidly decay to zero. In contrast with the case of SI model, the second peak in SIS model is less than the first peak, because the recovery mechanism slows down the propagation velocity of diseases, which reduces the variability of the prevalence. That is to say, the recovery mechanism reduces the variability of epidemic spreading. Another extremely obvious difference is the variability decreases to a steady value at the stationary state. For SIR model in Fig. 2(f), as time goes by, the epidemics has the greater and greater variability, which is caused by the different lifetimes of the epidemics in 2 × 10^4^ independent realizations.

### B. The local community

Considering the relative independence of a local community, we should take the prevalence and variability into account. On the other hand, since disease must be transmitted through bridgenesses from the first community to the second community, this study contributes to understand the effect of them on epidemic spreading.^52^ In this section, we will specifically analyze the effect of different distances of seeds (to the bridgeness in the first community) on epidemic spreading in the second community.

At first, the arrival time of disease is defined as the moment that infectious individual first occurs in the second community in each realization, thus the distribution of arrival time is obtained through massive realizations. In Fig. 3, the distribution of arrival time for the different initial seeds is shown. For SI model, the arrival distribution of the bridgeness as seed *d* = 0 strictly obey the distribution *P*(*t*) = *λ*(1 − *λ*)*^t^*^−1^. When disease seed is the node with one step to bridgeness, the arrival time increases generally, and the distribution becomes much wider and flatter. With the further increasing of distance of seed to the bridgeness (such as *d* = 3, 4), the distributions are nearly the same. It is understood that due to the finite size effect of network, the disease is transmitted through weak tie to the second community till overall outbreak happened in the first community. For SIS model, as a result of the recovery mechanism, the distributions of arrival time are much more evenly and smoothly than that for SI model, given the various initial seeds. Compared with Fig. 3(a), we can find that there are two peaks for SIS and SIR model with *d* = 1. This is because the bridgeness may be infected through two basic pathways: the bridgeness may be infected directly by the initial seed (i.e., its neighboring node) in *t* ≤ 1/*μ*; the other route is the transmission of infection from the other neighboring nodes when the disease outbreak in the first community, thus the second peak occurs at *t* ≈ 20 (see Fig. 2(b)). For SIR model, owing to the recovery mechanism, the prevalence might well disappear in the first community before arriving at the weak tie. Consequently, arrival rate (i.e., the area of the distribution) is less than one, and the peak value of the corresponding distribution is less than that for both SI and SIS model.

**FIG. 4.**
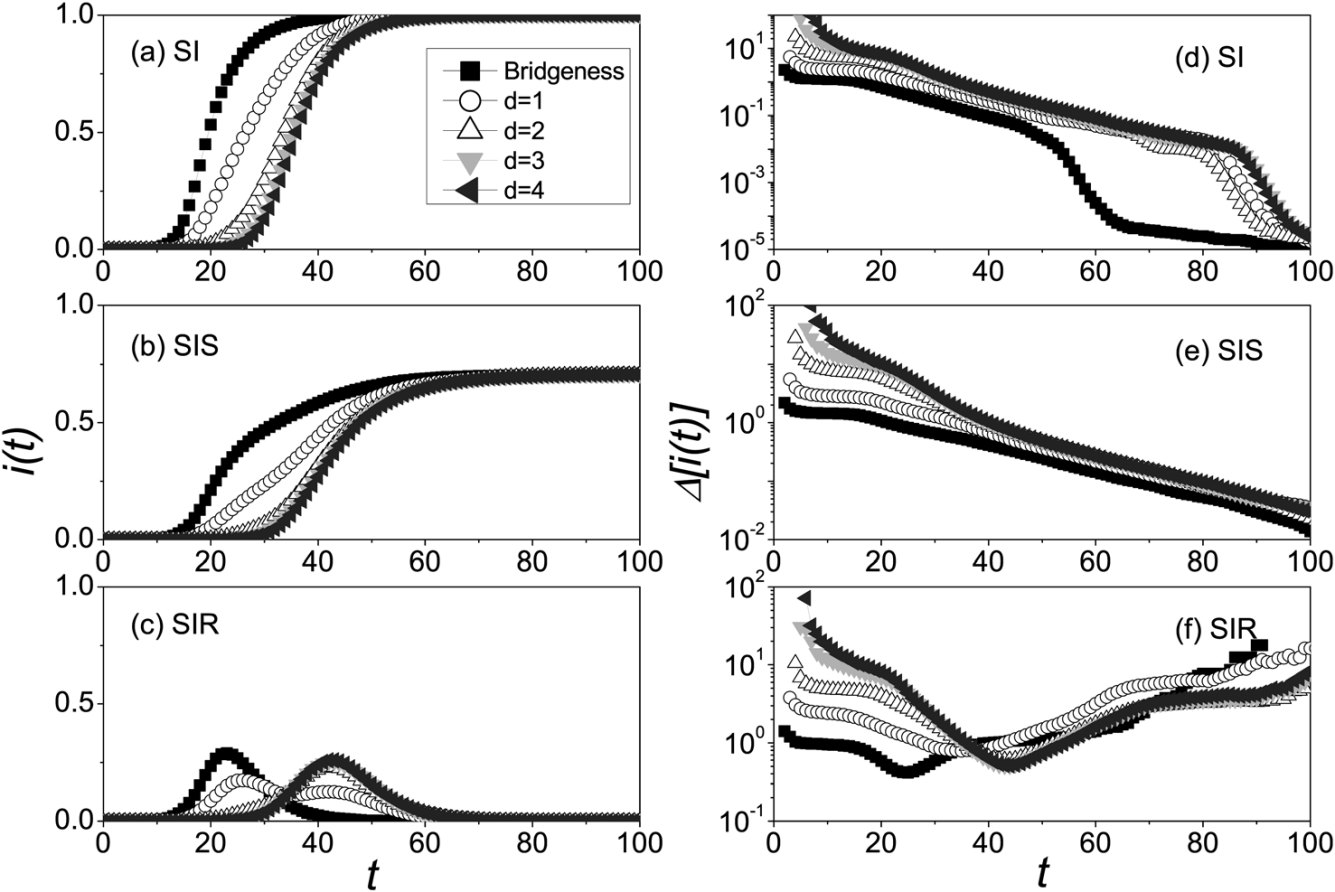
Evolution of both *i*(*t*) and Δ[i(t)] in the second community where the “squares,” “circles,” “triangleups,” “triangledowns,” and “trianglelefts” denote the cases of the bridgeness, *d* = 1, 2, 3, and 4, respectively. *i*(*t*) versus *t* for SI model (a), SIS model (b), and SIR model (c), and Δ[i(t)] versus *t* for SI model (d), SIS model (e), and SIR model (f). The results are averaged over 2 × 10^4^ independent realizations.

Fig. 4 shows the prevalence and variability in the second community which are generated from the different initial seeds in the first community. From Figs. 4(a)–4(c),we can easily know that the prevalence of the bridgeness acting as seed increases much faster than that in the other cases generated by the further seeds. In particular, for SIR model in Fig. 4(c), the peak of the prevalence (for the case of the bridgeness) occurs first and has the maximum value. In addition, there are two peaks of the prevalence for the case of *d* = 1, which is attributed to the propagation delay between two communities. Since there is only one interconnection between two communities, the chance of infecting one from the other is low. In other words, infection within intra-community will be much faster than inter-community infection. Therefore, the first peak reflects the outbreak and extinction of disease inside with infectious seed, and the second peak emerges after the other community is infected. Note that the two peaks can be only observed in SIR model because of the fact that there will be no peak if virus does not “die.” With the increase of distances *d*, the peak value is greater than that for *d* = 1, although the outbreaks occur later.

In Figs. 4(d)–4(f), it is a surprise that the variabilities in the second community are distinct from that in the global network. First, the variabilities in the second community are very large and also much greater than that in the global network in Fig. 2, which implies the huge unpredictability of prevalence produced in the local community. In particular, at the beginning of outbreaks, the variability for the case of *d* = 4 reaches about 50, which is 100 times the maximum value 0.5 in the global network. Second, the closer distance of the seed to bridgeness, the lower level of variability it has in the local community. In particular, the maximum variability value for the case of the bridgeness is only about 1, which is much less than 50 for the case of *d* = 4. Thus, the bridgeness plays a significant role in enhancing the predictability that the closer initial seed to the bridgeness, the more accurate the predictability is. Third, each curve of variabilities can be divided into four parts: the sudden drop stage, the relatively stable stage, the slowing-down stage, and the final stage of outbreaks (i.e., the exponential decay stage for SI model, the steady state stage for SIS model, and the sharp increase stage for SIR model, respectively). The first stage is originated from the uncertain arrival time of disease, with the increase of the arrival rate, the variability decreases. The second stage (10 < *t* < 20 in most cases) is induced by the interplay between the outbreak and the arrival of diseases in massive realizations: on one hand, the outbreaks in some realizations upgrade the variability; on the other hand, the increase of the arrival rate counteracts this effect. As the infection density is close to saturated, the variability will enter the slowing-down stage. What is noteworthy is that the minimum variability value just corresponds to the peak value of prevalence for SIR model. In the end of epidemic spreading, the variabilities Δ[i(t)] display the distinct phenomena for the different disease models; for instance, SIR model shows the higher and higher variabilities.

## V. CONCLUSIONS AND DISCUSSIONS

In conclusions, we have studied the variability of CP in complex networks and get the clear understanding that the different network structures can remarkably influence the prevalence and its variability. First, we find that the variability difference between homogeneous and heterogeneous networks is very narrow, although the heterogeneous structure induces a lighter prevalence. Second, two peaks of both the prevalence and variability are shown in the community network. It is noted that in the local community, the extraordinarily large variability in early stage of the outbreak makes the prediction of disease spreading hard. This result is in accordance with Ref. 53 in which the networks with strong community structures are of weak synchronizability, and the amplitudes of the time series in the local communities are much larger than that in the global networks. Fortunately, the bridgeness plays a significant role in enhancing the predictability, the closer initial seed to the bridgeness, the more accurate the predictability is. This result suggests that bridgenesses may be the ideal detection stations in community networks. Moreover, the different reaction mechanisms of disease models can result in the distinct variabilities. Especially for the case of SIR model, the greater and greater variabilities are observed at the end of the epidemics for the different lifetimes of the epidemics in various realizations.

The community network employed in this study is much more simple, but the actual community networks have complex structures, such as multifarious communities, many bridgenesses, and the heterogeneous degree distribution in a local community. Therefore, the further investigation should be focused on the more complex community networks.

## References

[1] N. T. J. Bailey, The Mathematical Theory of Infectious Diseases, 2nd ed. (Griffin, London, 1975).

[2] R. M. Anderson and R. M. May, Infectious Disease of Humans (Oxford University Press, Oxford, 1992).

[3] O. Diekmann and J. A. P. Heesterbeek, Mathematical Epidemiology of Infectious Diseases: Model Building, Analysis and Interpretation (Wiley, New York, 2000).

[4] D. J. Dailey and J. Gani, Epidemic Modelling: An Introduction (Cambridge University Press, Cambridge, 2001).

[5] N. M. Ferguson, M. J. Keeling, W. J. Edmunds, R. Gani, B. T. Greenfell, and R. M. Anderson, Nature 425, 681 (2003). 10.1038/nature02007 14562094 PMC7095314

[6] R. Albert and A.-L. Barabási, Rev. Mod. Phys. 74, 47 (2000). 10.1103/RevModPhys.74.47

[7] R. Cohen, K. Erez, D. ben-Avraham, and S. Havlin, Phys. Rev. Lett. 85, 4626 (2000). 10.1103/PhysRevLett.85.4626 11082612

[8] R. Pastor-Satorras and A. Vespignani, Phys. Rev. Lett. 86, 3200 (2001). 10.1103/PhysRevLett.86.3200 11290142

[9] R. Pastor-Satorras and A. Vespignani, Phys. Rev. E 63, 066117 (2001). 10.1103/PhysRevE.63.066117 11415183

[10] R. M. May and A. L. Lloyd, Phys. Rev. E 64, 066112 (2001). 10.1103/PhysRevE.64.066112

[11] A. L. Lloyd and R. M. May, Science 292, 1316 (2001). 10.1126/science.1061076 11360990

[12] V. M. Eguíluz and K. Klemm, Phys. Rev. Lett. 89, 108701 (2002). 10.1103/PhysRevLett.89.108701 12225235

[13] M. Boguñá and R. Pastor-Satorras, Phys. Rev. E 66, 047104 (2002). 10.1103/PhysRevE.66.047104 12443385

[14] M. Boguñá, R. Pastor-Satorras, and A. Vespignani, Phys. Rev. Lett. 90, 028701 (2003). 10.1103/PhysRevLett.90.028701 12570587

[15] M. Barthélemy, A. Barrat, R. Pastor-Satorras, and A. Vespignani, J. Theory Biol. 235, 275 (2005). 10.1016/j.jtbi.2005.01.011 15862595

[16] M. Tang, Z. Liu, and J. Zhou, Phys. Rev. E 74, 036101 (2006); 10.1103/PhysRevE.74.036101 17025702

[17] V. Kishore, M. S. Santhanam, and R. E. Amritkar, Phys. Rev. Lett. 106, 188701 (2011). 10.1103/PhysRevLett.106.188701 21635132

[18] V. Colizza, A. Barrat, M. Barthlemy, and A. Vespignani, Proc. Natl. Acad. Sci. U.S.A. 103, 2015 (2006). 10.1073/pnas.0510525103 16461461 PMC1413717

[19] P. Crépey, F. P. Alvarez, and M. Barthélemy, Phys. Rev. E 73, 046131 (2006). 10.1103/PhysRevE.73.046131 16711902

[20] A. Gautreau, A. Barrat, and M. Barthélemy, J. Stat. Mech. 2007, L09001 10.1088/1742-5468/2007/09/L09001

[21] A. Gautreau, A. Barrat, and M. Barthélemy, J. Theory Biol. 251, 509 (2008). 10.1016/j.jtbi.2007.12.001 18222486

[22] M. Tang, L. Liu, and Z. Liu, Phys. Rev. E 79, 016108 (2009). 10.1103/PhysRevE.79.016108 19257108

[23] M. Tang, L. Liu, and B. Li, Europhys. Lett. 87, 18005 (2009). 10.1209/0295-5075/87/18005

[24] M. Barthélemy, C. Godrèche, and J.-M. Luck, J. Theory Biol. 267, 554 (2010). 10.1016/j.jtbi.2010.09.015 20863838

[25] Z. Liu and B. Hu, Europhys. Lett. 72(2), 315 (2005). 10.1209/epl/i2004-10550-5

[26] L. Huang, K. Park, and Y.-C. Lai, Phys. Rev. E 73, 035103–R (2006). 10.1103/PhysRevE.73.035103 16605585

[27] Y. Zhou, Z. Liu, and J. Zhou, Chin. Phys. Lett. 24, 581 (2007). 10.1088/0256-307X/24/2/078

[28] X. Wu and Z. Liu, Physica A 387, 623 (2008). 10.1016/j.physa.2007.09.039

[29] X. Chu, J. Guan, Z. Zhang, and S. Zhou, J. Stat. Mech. 2009, P07043 10.1088/1742-5468/2009/07/P07043

[30] D. Chen, Y. Fu, and M. Shang, Physica A 388, 2741 (2009). 10.1016/j.physa.2009.03.022

[31] W. Huang and C. Li, J. Stat. Mech. 2007, P01014 10.1088/1742-5468/2007/01/P01014

[32] T. Zhou, J.-G. Liu, W.-J. Bai, G.-R. Chen, and B.-H. Wang, Phys. Rev. E 74, 056109 (2006). 10.1103/PhysRevE.74.056109 17279970

[33] C. Castellano and R. Pastor-Satorras, Phys. Rev. Lett. 96, 038701 (2006). 10.1103/PhysRevLett.96.038701 16486782

[34] M. Ha, H. Hong, and H. Park, Phys. Rev. Lett. 98, 029801 (2007). 10.1103/PhysRevLett.98.029801 17358660

[35] C. Castellano and R. Pastor-Satorras, Phys. Rev. Lett. 98, 029802 (2007). 10.1103/PhysRevLett.98.029802

[36] H. Hong, M. Ha, and H. Park, Phys. Rev. Lett. 98, 258701 (2007). 10.1103/PhysRevLett.98.258701 17678061

[37] R. Yang, B.-H. Wang, J. Ren, W.-J. Bai, Z.-W. Shi, W.-X. Wang, T. Zhou, Phys. Lett. A 364, 189 (2007). 10.1016/j.physleta.2006.12.021

[38] R. Yang, T. Zhou, Y.-B. Xie, Y.-C. Lai, and B.-H. Wang, Phys. Rev. E 78, 066109 (2008). 10.1103/PhysRevE.78.066109 19256907

[39] R. Yang, L. Huang, and Y.-C. Lai, Phys. Rev. E 78, 026111 (2008). 10.1103/PhysRevE.78.026111 18850901

[40] J. D. Noh and H. Park, Phys. Rev. E 79, 056115 (2009). 10.1103/PhysRevE.79.056115 19518529

[41] S. H. Lee, M. Ha, H. Jeong, J. D. Noh, and H. Park, Phys. Rev. E 80, 051127 (2009). 10.1103/PhysRevE.80.051127 20364967

[42] M. A. Muñoz, R. Juhász, C. Castellano, and G. Ódor, Phys. Rev. Lett. 105, 128701 (2010). 10.1103/PhysRevLett.105.128701 20867681

[43] P. Erdős and A. Rényi, Publ. Math. Inst. Hung. Acad. Sci. 5, 17 (1960).

[44] A.-L. Barabási and R. Albert, Science 286, 509 (1999). 10.1126/science.286.5439.509 10521342

[45] R. Albert and A.-L. Barabási, Rev. Mod. Phys. 74, 47 (2002). 10.1103/RevModPhys.74.47

[46] M. E. J. Newman, Phys. Rev. Lett. 89, 208701 (2002); 10.1103/PhysRevLett.89.208701 12443515

[47] See http://www.facebook.com.

[48] See http://www.youtube.com.

[49] See http://www.xiaonei.com.

[50] J.-P. Onnela, J. Saramäki, J. Hyvönen, G. Szabó, D. Lazer, K. Kaski, J. Kertész, and A.-L. Barabási, Proc. Natl. Acad. Sci. U.S.A. 104, 7332 (2007). 10.1073/pnas.0610245104 17456605 PMC1863470

[51] X.-Q. Cheng, F.-X. Ren, H.-W. Shen, Z.-K. Zhang, and T. Zhou, J. Stat. Mech. 2010, P10011 10.1088/1742-5468/2010/10/P10011

[52] J. Zhao, J. Wu, and K. Xu, Phys. Rev. E 82, 016105 (2010). 10.1103/PhysRevE.82.016105 20866687

[53] G. Yan, Z.-Q. Fu, J. Ren, and W.-X. Wang, Phys. Rev. E 75, 016108 (2007). 10.1103/PhysRevE.75.016108 17358225

